# Critical thinking in occupational therapy education: A mixed‐methods study

**DOI:** 10.1111/1440-1630.70108

**Published:** 2026-07-08

**Authors:** Laura Irvine‐Brown, Ana Malfitano, Shawna Mastro Campbell, Amelia Di Tommaso, Lisette Farias

**Affiliations:** ^1^ School of Health Sciences and Social Work Griffith University Gold Coast Queensland Australia; ^2^ Postgraduate Program in Occupational Therapy, Occupational Therapy Department Federal University of São Carlos São Carlos Brazil; ^3^ School of Psychology, Faculty of Society and Design Bond University Gold Coast Queensland Australia; ^4^ Griffith University Gold Coast Queensland Australia; ^5^ School of Allied Health Science and Human Performance Adelaide University Adelaide South Australia Australia; ^6^ Division of Occupational Therapy, Department of Neurobiology, Care Sciences and Society Karolinska Institutet Stockholm Sweden

**Keywords:** educators, epistemology, higher education, occupational therapists

## Abstract

**Introduction:**

Critical thinking is widely recognised as essential for occupational therapy education and practice, yet its conceptualisation, teaching, and assessment remain inconsistent across international contexts. Existing literature highlights a lack of clarity regarding effective pedagogical approaches and a limited understanding of how educators make sense of critical thinking within occupational therapy curricula. This study examined how occupational therapy educators across different countries conceptualise critical thinking and how it features in the programmes they teach.

**Methods:**

A convergent mixed‐methods design was used. Occupational therapy educators completed an online survey (available in Spanish, Portuguese, and English) containing 28 quantitative items and six open‐ended qualitative questions. Quantitative data were analysed using descriptive statistics, and group comparisons were made using the Kruskal–Wallis and Dunn tests. Qualitative data underwent inductive content analysis within language groups and were later synthesised across datasets through reflexive, collaborative analysis.

**Consumer and Community Involvement:**

No consumers were involved in the study conceptualisation or analysis.

**Results:**

Responses revealed a spectrum of conceptualisations of critical thinking, ranging from concrete, pragmatic, and clinically focused understandings to abstract, socially oriented, and transformative perspectives. English‐language responses tended to emphasise clinical reasoning, evidence use, and decision‐making, whereas Portuguese and Spanish responses more strongly foregrounded reflection on knowledge, sociopolitical structures, and critical action. Teaching practices mirrored this spectrum: Critical thinking was taught unevenly across curricula, most commonly within clinical reasoning or foundational occupational therapy subjects, with fewer programmes explicitly addressing broader sociocultural or transformative dimensions.

**Conclusion:**

Findings highlight significant epistemological variation shaping how critical thinking is conceptualised and enacted in occupational therapy education. Recognising these epistemological foundations, rather than seeking a universal definition, may support more intentional, inclusive, and socially responsive curriculum design.

## PLAIN LANGUAGE SUMMARY

Critical thinking is an important skill for occupational therapists, helping them question assumptions and understand how social, cultural, and political factors influence people's everyday lives. However, occupational therapy educators around the world may not always agree on what critical thinking means or how it should be taught to students. This study explored how occupational therapy educators from different countries understand critical thinking and how it features in their university programmes. Educators completed an online survey with multiple‐choice questions and open‐ended questions. We analysed the numerical answers using statistics and the written answers using qualitative content analysis, comparing similarities and differences across language groups.

We found that educators think about critical thinking in two main ways. Some described it as a practical, concrete process linked to clinical reasoning, evidence evaluation, and intervention decision‐making. These views were most common in English‐language responses. Others described critical thinking as more abstract or as part of a social process. This included questioning how knowledge is created, considering social and political influences on occupation and everyday life, and taking action to address injustices. These views were more common in Portuguese and Spanish responses. Educators also differed in perceptions of how and where critical thinking featured in their programmes. For many, it featured most often in clinical or professional reasoning subjects. A smaller number reported that it featured in courses about society, culture, or social justice. The study shows that critical thinking is shaped by different cultural, epistemological, and educational traditions. Becoming more aware of these differences can help occupational therapy educators design more inclusive and socially responsive curricula.

**Key Points for Occupational Therapy**

- Addressing uneven teaching across curricula can strengthen students' capacity for comprehensive critical thinking.
- Recognising the spectrum of critical thinking supports more adaptable, context‐responsive occupational therapy practice.
- Occupational therapy benefits from recognising diverse epistemologies shaping critical thinking and practice.

## 1 INTRODUCTION

Wittman and Velde (2002) asked educators: ‘How can we facilitate the promotion of critical thinking skills that will prepare our students to practice effectively in a multicultural, global world?’ (p. 456). Two decades later, this question remains central to professional preparedness amid evolving social needs, artificial intelligence, and emerging diseases and disasters. Critical thinking is widely regarded as essential for developing responsible professionals who can reflect on and evaluate diverse perspectives and understand the links between local and global events (Behar‐Horenstein & Niu, 2011; Kraska et al., 2018). Accordingly, critical thinking has become a key goal of higher education institutions worldwide (Liyanage et al., 2021) and in occupational therapy (Gilfillan et al., 2024; Lederer, 2007).

Several literature reviews on teaching critical thinking in higher education and in occupational therapy reflect the importance of this topic for researchers and educators (Berg et al., 2023; Pitonyak et al., 2020). Although these reviews identify strategies such as case studies and role‐playing as beneficial for supporting students' development of critical thinking in the classroom, evidence for their effectiveness in occupational therapy remains inconclusive. For instance, a few studies show that occupational therapy students can benefit from instruction designed to develop the disposition to think critically (Allen & Toth‐Cohen, 2019; Lederer, 2007). Yet, the literature still lacks clarity regarding which instructional methods, learning outcomes, or assessment approaches most effectively foster critical thinking in occupational therapy education. This gap is problematic because students and educators may hold different expectations regarding how critical thinking should be demonstrated and assessed. Such inconsistencies may contribute to uneven integration of critical thinking across programmes (Gilfillan et al., 2024).

Critical thinking is often included in occupational therapy curriculum as a learning outcome, yet there is a lack of evaluation tools to demonstrate whether students have developed these skills (Pitonyak et al., 2020). Critical thinking is also frequently linked to broader professional outcomes, such as reflective practice, ethical decision‐making, and cultural competence, rather than being conceptualised as a distinct construct (Pitonyak et al., 2020). Studies on the topic emphasised the importance of supporting students' active engagement in their learning, as opposed to traditional pedagogies that position students in a more passive role (Lederer, 2007). Despite these insights, the evidence base in occupational therapy remains small and fragmented, and methodologically limited, restricting the field's ability to establish shared expectations or best practices for teaching critical thinking. This underscores the need for more studies in occupational therapy education that employ diverse research designs (Pitonyak et al., 2020) to explore educators' conceptualisations of critical thinking and its enactment in teaching. Such work would help advance the field beyond the limited best‐practice guidance currently available (Berg et al., 2023).

Recent scholarship also highlights that educators' understanding of critical thinking varies considerably (Gilfillan et al., 2024; Liyanage et al., 2021). This variation underscores the need to develop clearer definitions of critical thinking and to identify effective methods to help students challenge their assumptions and biases (Berg et al., 2023; Pitonyak et al., 2020). Moreover, it has become increasingly important to distinguish critical thinking from clinical reasoning to establish it as a unique and explicit construct in occupational therapy education (Gilfillan et al., 2024). To address these gaps, research must enhance international collaboration to explore how critical thinking is understood and enacted across diverse educational contexts.

The purpose of this study was to use both a quantitative and a qualitative approach to examine how occupational therapy educators across different countries conceptualise critical thinking and how it features within their programmes and courses. To achieve this, the study aimed to answer the following questions:

1. What conceptualisations of critical thinking are used by occupational therapy educators across different countries, and how do they teach and assess critical thinking within their educational programmes?
2. How do occupational therapy academics experience and make sense of the ways they conceptualise and teach critical thinking within their educational programmes?

## 2 METHODS

### 2.1 Ethics

All researchers involved had a master's or PhD degree when conducting the study and worked at universities across different countries. One of the authors, outside of occupational therapy, was consulted regarding the statistics employed to conduct the quantitative data analysis. Electronic informed consent was completed with all eligible participants recruited.

### 2.2 Study design

A convergent mixed‐methods design was utilised (Creswell & Clark, 2007). Results from a study using an online survey (Research Question 1) were convergent with open‐ended questions from the same survey (Research Question 2) to compare and contrast the findings. This method aimed to gather complementary data on the same topic across various countries.

### 2.3 Participants and recruitment

As inclusion criteria, participants were eligible if they were occupational therapists currently employed as educators in an occupational therapy programme and could respond to the survey in English, Spanish, or Portuguese. Participants were recruited through social media (Facebook flyers and Twitter posts), advertising on the World Federation of Occupational Therapists (WFOT) and Occupational Therapy Australia (OTA) research website platforms, advertising on the Occupational Therapy International Online Network (OTION) evidence‐based research forums, and via email invitations through the authors' professional networks. Additionally, 731 email addresses for ‘Head of Program’ of WFOT‐accredited programmes were collated from the WFOT public website. An invitation to participate was sent to each of these email addresses, with a request to distribute it among staff.

All recruitment posts and emails contained a link to the online survey, which started with a participant information and consent form. Participants had to provide consent before accessing the survey. At the completion of the survey, participants were given the opportunity to register their interest in participating in an online focus group as Phase Two of the study (the results of Phase Two are published elsewhere).

### 2.4 Positionality statement

Our research team consists of cisgender, female, working‐class‐background occupational therapy educators and occupational science academics working across universities in Brazil, Australia and Sweden. Our ethnicities vary from Mestiza, Latina, and White. One author is a registered psychologist, clinical psychology academic, and educator. These identities, informed by work in areas such as community development, social justice, and health equity, shape how we understand and investigate critical thinking in occupational therapy curricula. To manage our varied identities during the study, we engaged in ongoing team reflexivity to consider how our professional positions and shared commitments might shape the development of research questions, methodological choices, and interpretations.

### 2.5 Data collection tools

Quantitative data were collected via an online survey questionnaire. Qualitative data were obtained from open‐ended questions included in the survey.

#### 2.5.1 Questionnaire

An online questionnaire was developed in LimeSurvey® to gather data on how occupational therapy academics conceptualise, teach, and assess critical thinking. Three authors collaboratively drafted the initial English‐language survey items to address the research questions, after which a fourth author reviewed and refined the wording. The questionnaire was then piloted with five occupational therapy academics, each with English as a first, second, or third language, from five different countries. The pilot assessed content and internal validity, clarity of questions, and international applicability. Minor revisions were made based on this feedback. The final survey included 28 items across three sections: (1) demographic information; (2) conceptualisations of critical thinking in occupational therapy and occupational therapy education; and (3) agreement with and perceived alignment between participants' programmes and a proposed definition of critical thinking. This third section introduced the following definition of critical thinking in occupational therapy education:

We define critical thinking in occupational therapy education from the perspective of criticality theorists (Barnett, 1997; Davies, 2015) as the combination of critical reason, critical reflection, and critical action. In this sense, we conceptualise critical thinking as including the technical skills in analysis, evaluation, and logical argumentation (critical reason); the reflective skills and dispositions to question self, knowledge, and the wider world (critical reflection); and one's participation in society as a critically engaged citizen‐in‐the‐world, seeking to question taken‐for‐granted assumptions and transform inequitable power structures and practices (critical action). This critical thinking problematises practice and theory and connects reflection and action. A critical thinker does more than reason; they also act in moral, ethical, and just ways on the basis of their reasoned judgements and in pursuit of social justice.

Participants then responded to items relating to this definition. Most survey items (n = 22) were closed‐ended, comprising one yes/no question, ten multiple‐choice questions, and eleven 5‐point Likert‐scale questions ranging from *not at all* to *a large extent*.

#### 2.5.2 Open‐ended questions

Six open‐ended questions were included, with no word limit after the survey items. These questions were as follows:

- Does your programme have specific subjects that explicitly teach critical thinking (CT)? If so, please add the names of these here: (Comment box).
- Please explain the reason for your above rating (extent CT essential for OT).
- Why do you think CT might be useful in OT education?
- Please describe how you conceptualise/define CT in OT education.
- To what extent do you agree with this definition of CT? Why/why not?
- Please provide examples of teaching methods you use to develop students' critical thinking.

To ensure linguistic accuracy and enhance international validity, the survey was translated into Spanish and Portuguese using a back‐translation approach. AM and LF independently translated the English survey into Portuguese and Spanish, respectively. These versions were then reviewed against the original English survey by native English speakers with academic proficiency in Portuguese and Spanish, external to the research team. Only minimal refinements were required.

### 2.6 Data analysis

#### 2.6.1 Quantitative

Data were exported from LimeSurvey® into the Statistical Package for the Social Sciences (SPSS) version 29 for analysis. Qualitative responses were removed at this stage, and quantitative data were manually coded before being examined using descriptive statistics. Based on insights generated from the qualitative analysis, additional comparative analyses were conducted to explore potential differences between language groups. Language group medians were evaluated for differences by conducting a Kruskal–Wallis test, followed by Dunn's post hoc pairwise comparisons to identify where significant differences occurred.

#### 2.6.2 Qualitative

Given the volume of qualitative data and the variability in response length, including single‐sentence and one‐word answers, inductive content analysis (Graneheim & Lundman, 2004) was selected as the most appropriate analytic approach. Qualitative responses were first separated by language and then organised according to the research questions they addressed. Four questions explored participants' conceptualisations of critical thinking in occupational therapy, and two questions examined how they teach it.

To support rigour and linguistic accuracy, authors analysed qualitative responses in their first language. As the majority of responses (59.3%) were in English, two authors (LIB and AdT), both native English speakers, independently analysed the English data. Each author reviewed the data separately, documented initial codes in individual files, and subsequently met to compare and discuss their analyses, confirming consistency between coding patterns. Reflexivity was integrated throughout the process, with both authors recording key reflections, assumptions, and analytical decisions. A second round of analysis was conducted to quantify prominent codes, including the frequency with which specific ideas or concepts appeared across participants' responses.

This analytic approach was then shared with the Portuguese‐ and Spanish‐speaking researchers, who independently conducted the same round of content analysis on the qualitative data in their respective languages. Each researcher produced an English‐language list of codes derived from their dataset, accompanied by selected translated quotations to provide contextual grounding for the coding decisions. Subsequently, a series of meetings was held with one researcher from each language group to discuss and compare the emerging codes, with the intention of analysing the qualitative data collectively, in line with the aims of the study. During these discussions, nuanced differences related to language and cultural interpretation were noted and explored. Following this collaborative process, all codes generated from the English, Spanish, and Portuguese datasets were compiled into a single document, while remaining organised by language group.

The first author then compared the three sets of codes to identify similarities and differences, synthesising these into preliminary thematic findings. Throughout this stage, she engaged in ongoing reflexive review of the English‐language data to ensure that the developing interpretations remained grounded in the raw responses. The resulting findings were then shared with the other researchers, who independently verified them against the original Portuguese and Spanish qualitative data they had analysed.

## 3 RESULTS

### 3.1 Participants

A total of 118 participants completed the survey (see Table 1). Most were aged 35–44 (32%) and held a PhD (55%) as their highest qualification. Most had been teaching in occupational therapy programmes for over 7 years (72%). The majority taught in WFOT‐approved (85%) entry‐level programmes (76%) lasting 4–4.5 years (34%). Most programmes enrolled between 100 and 200 students (29%) or 200 and 300 students (26%). Participants represented 29 countries, with significant numbers from Europe, Latin America, and North America. Other countries included Mexico, Colombia, India, Israel, Jordan, Tunisia, Kenya, Zimbabwe, and the Philippines.

**TABLE 1:** Participant demographics.

Participant demographics	n (%)
Language in which the survey was completed	
English	70 (59.3)
Portuguese	26 (22)
Spanish	22 (18.6)
Age	
25–34	19 (16.1)
35–44	38 (32.2)
45–54	28 (23.7)
55–64	30 (25.4)
65–74	3 (2.5)
Highest level of education	
Bachelor's degree	8 (6.8)
Graduate certificate/diploma	5 (4.2)
Master's degree	40 (33.9)
PhD or above	65 (55.1)
Years as an occupational therapist	
Less than 1	2 (1.7)
1–3	5 (4.2)
4–6	7 (5.9)
7–9	11 (9.3)
More than 10	93 (78.8)
Years working in occupational therapy higher education	
Less than 1	5 (4.2)
1–3	11 (9.3)
4–6	16 (13.6)
7–9	18 (15.3)
More than 10	68 (57.6)

Programme demographics		n (%)
WFOT‐approved programme?		
Yes		101 (85.6)
No		17 (14.4)
World region	Country	
Latin and Central America	Argentina	5 (4.2)
	Brazil	27 (22.9)
	Chile	6 (5.1)
	Colombia	6 (5.1)
	Mexico	1 (0.8)
Asia	Philippines	1 (0.8)
	India	2 (1.7)
	Israel	1 (0.8)
	Jordan	1 (0.8)
Oceania	Australia	12 (10.2)
	New Zealand	2 (1.7)
Europe	Austria	3 (2.5)
	Denmark	2 (1.7)
	Spain	1 (0.8)
	France	5 (4.2)
	Germany	1 (0.8)
	Malta	1 (0.8)
	Netherlands	2 (1.7)
	Portugal	2 (1.7)
	Sweden	4 (3.4)
	Switzerland	2 (1.7)
	United Kingdom	11 (9.3)
North America	Canada	7 (5.9)
	United States	8 (6.8)
Africa	Kenya	2 (1.7)
	South Africa	1 (0.8)
	Tunisia	1 (0.8)
	Zimbabwe	1 (0.8)
Entry level or graduate?		
Entry level		76 (64.4)
Graduate entry level		19 (16.1)
Both		23 (19.5)
Years of full‐time study		
2–2.5		26 (22)
3–3.5		30 (25.4)
4–4.5		41 (34.7)
5 or more		21 (17.8)
Number of students in the programme		
0–100		26 (22)
100–200		35 (29.7)
200–300		31 (26.3)
300–400		13 (11)
400–500		4 (3.4)
More than 500		9 (7.6)

Abbreviation: WFOT, World Federation of Occupational Therapists.

### 3.2 Quantitative results

#### 3.2.1 Conceptualising critical thinking

Overall, all participants concurred that critical thinking is integral to effective occupational therapy practice, with 90.7% indicating a significant extent and 9.3% indicating a moderate extent (see Table 2). The majority also expressed agreement with the proposed definition of critical thinking, to a large extent (90.7%) or to a moderate extent (28.8%). No statistically significant differences in responses were observed across different language groups (see Table 3).

**TABLE 2:** Pooled quantitative data of responses to Likert‐scale questions.

Question		Not at all	Small extent	Somewhat	Moderate extent	Large extent	Not sure	N/A
		n (%)	n (%)	n (%)	n (%)	n (%)	n (%)	
To what extent do you think critical thinking is essential for effective occupational therapy practice?		0 (0)	0 (0)	0 (0)	11 (9.3)	107 (90.7)	—	—
To what extent do you think critical thinking is valued in the OT programme you teach in?		0 (0)	11 (9.3)	27 (22.9)	41 (34.7)	39 (33.1)	—	—
To what extent do you agree with this type^a^ of critical thinking?		0 (0)	1 (0.8)	3 (2.5)	34 (28.8)	80 (67.8)	—	—
To what extent do you feel this type^a^ of critical thinking features in your:	Programme	1 (0.8)	15 (12.7)	29 (24.6)	39 (33.1)	32 (27.1)	2 (1.7)	—
	Course/s you teach	1 (0.8)	8 (6.8)	19 (16.1)	29 (24.6)	59 (50)	2 (1.7)	—
To what extent do you feel this type^a^ of critical reflection features in your:	Programme	1 (0.8)	19 (16.1)	32 (27.1)	33 (28)	31 (26.3)	2 (1.7)	—
	Course/s you teach	1 (0.8)	9 (7.6)	18 (15.3)	37 (31.4)	51 (43.2)	2 (1.7)	—
To what extent do you feel this type^a^ of critical reason features in your:	Programme	3 (2.5)	14 (11.9)	27 (22.9)	44 (37.3)	28 (23.7)	2 (1.7)	—
	Course/s you teach	1 (0.8)	8 (6.8)	21 (17.8)	45 (38.1)	42 (35.6)	1 (0.8)	—
To what extent do you feel this type^a^ of critical action features in your:	Programme	3 (2.5)	26 (22)	31 (26.3)	39 (33.1)	17 (14.4)	2 (1.7)	—
	Course/s you teach	5 (4.2)	18 (15.3)	18 (15.3)	38 (32.2)	36 (30.5)	3 (2.5)	—
To what extent do you feel this type^a^ of critical reflection features in the following teaching activities:	Class‐based teaching	2 (1.7)	13 (11)	23 (19.5)	41 (34.7)	36 (30.5)	3 (2.5)	0 (0)
	Simulation	7 (5.9)	12 (10.2)	32 (27.1)	26 (22)	15 (12.7)	13 (11)	13 (11)
	Practice education	3 (2.5)	7 (5.9)	19 (16.1)	32 (27.1)	42 (35.6)	11 (9.3)	4 (3.4)
To what extent do you feel this type^a^ of critical reason features in the following teaching activities:	Class‐based teaching	1 (0.8)	15 (12.7)	22 (18.6)	35 (29.7)	40 (33.9)	5 (4.2)	0 (0)
	Simulation	7 (5.9)	17 (14.4)	27 (22.9)	25 (21.2)	19 (16.1)	12 (10.2)	11 (9.3)
	Practice education	2 (1.7)	13 (11)	16 (13.6)	38 (32.2)	32 (27.1)	13 (11)	4 (3.4)
To what extent do you feel this type^a^ of critical action features in the following teaching activities:	Class‐based teaching	4 (3.4)	25 (21.2)	24 (20.3)	36 (30.5)	28 (23.7)	1 (0.8)	0 (0)
	Simulation	10 (8.5)	27 (22.9)	25 (21.2)	17 (14.4)	15 (12.7)	12 (10.2)	12 (10.2)
	Practice education	5 (4.2)	13 (11)	30 (25.4)	28 (23.7)	25 (21.2)	12 (10.2)	5 (4.2)
To what extent has completing this survey supported your awareness of the way you think about CT in OT education?		4 (3.4)	5 (12.7)	32 (27.1)	38 (32.2)	25 (21.2)	4 (3.4)	118 (100)

*Note*: Colour intensity indicates frequency of response: lightest colour 0%–20%, second lightest colour 21%–40%, middle colour 41%–60%, second darkest colour 61%–80%, and darkest colour 81%–100%.

Abbreviations: N/A, not applicable; OT, occupational therapy.

^a^ Definitions were provided for each of these terms.

**TABLE 3:** Language group differences.

Question		English (n = 70) Median (IQR)	Portuguese (n = 26) Median (IQR)	Spanish (n = 22) Median (IQR)	H statistic (df)	Differences
To what extent do you think critical thinking is essential for effective occupational therapy practice?		5.0 (0.0)	5.0 (0.0)	4.0 (2.0)	1.76 (2)	—
To what extent do you think critical thinking is valued in the OT programme you teach into?		4.0 (2.0)	4.0 (1.0)	4.0 (2.0)	1.85 (2)	—
To what extent do you agree with this type^a^ of critical thinking?		5.0 (1.0)	5.0 (1.0)	5.0 (0.0)	2.44 (2)	—
To what extent do you feel this type^a^ of critical thinking features in your:	Programme	3.0 (2.0)	4.0 (1.0)	4.0 (2.0)	1.60 (2)	—
	Course/s you teach	4.0 (2.0)	5.0 (1.0)	5.0 (1.0)	6.49 (2)*	P > E (P = 0.04) S > E (P = 0.05)
To what extent do you feel this type^a^ of critical reflection features in your:	Programme	3.0 (2.0)	4.0 (1.0)	4.0 (2.0)	0.92 (2)	—
	Course/s you teach	4.0 (2.0)	5.0 (1.0)	4.5 (1.0)	7.40 (2)*	P > E (P = 0.01)
To what extent do you feel this type^a^ of critical reason features in your:	Programme	4.0 (1.0)	3.5 (2.0)	3.5 (2.0)	2.14 (2)	—
	Course/s you teach	4.0 (2.0)	5.0 (1.0)	4.0 (1.0)	6.52 (2)*	P > E (P = 0.02)
To what extent do you feel this type^a^ of critical action features in your:	Programme	3.0 (2.0)	4.0 (2.0)	3.5 (2.0)	6.40 (2)*	P > E (P = 0.02)
	Course/s you teach	3.0 (2.0)	5.0 (1.0)	4.5 (1.0)	32.06 (2)***	P > E (P = 0.001) S > E (P = 0.004)
To what extent do you feel this type^a^ of critical reflection features in the following teaching activities:	Class‐based teaching	4.0 (1.0)	4.0 (2.0)	4.0 (2.0)	0.42 (2)	—
	Simulation	3.0 (2.0)	3.0 (1.0)	3.0 (3.0)	0.03 (2)	—
	Practice education	4.0 (2.0)	4.5 (2.0)	4.5 (1.0)	4.02 (2)	—
To what extent do you feel this type^a^ of critical reason features in the following teaching activities:	Class‐based teaching	4.0 (2.0)	4.0 (2.0)	4.0 (2.0)	0.24 (2)	—
	Simulation	3.0 (2.0)	3.0 (1.0)	4.0 (3.0)	0.23 (2)	—
	Practice education	4.0 (1.0)	4.0 (2.0)	4.0 (1.0)	2.19 (2)	—
To what extent do you feel this type^a^ of critical action features in the following teaching activities:	Class‐based teaching	3.0 (2.0)	4.0 (2.0)	4.0 (2.0)	14.01 (2)***	P > E (P = 0.001) S > E (P = 0.01)
	Simulation	3.0 (1.0)	3.0 (2.0)	4.0 (3.0)	11.58 (2)**	P > E (P = 0.001) S > E (P = 0.05)
	Practice education	3.0 (2.0)	4.0 (2.0)	4.0 (2.0)	22.21 (2)***	P > E (P < 0.001) S > E (P = 0.004)
To what extent has completing this survey supported your awareness of the way you think about CT in OT education?		4.0 (1.0)	5.0 (1.0)	5.0 (1.0)	14.14 (2)***	P > E (P = 0.01) S > E (P = 0.002)

Abbreviations: CT, critical thinking; E, English; IQR, interquartile range; OT, occupational therapy; P, Portuguese; S, Spanish.

^a^ Definitions were provided.

* P < 0.05.

** P < 0.01.

*** P < 0.001.

#### 3.2.2 Teaching and learning activities for critical thinking

Table 3 reports the median and interquartile range for the three language groups, as well as comparisons of each language group's median using the Kruskal–Wallis test with Dunn's post hoc pairwise comparisons. In relation to teaching activities for critical thinking, participants believed that class‐based teaching and practical education experiences featured the various components of critical thinking (reflection, action, and reason) to a greater extent than simulated experiences. Regarding the extent to which critical action was featured in the three types of learning activities, Portuguese and Spanish responses indicated its presence to a significantly greater extent compared to English responses.

#### 3.2.3 Assessing critical thinking

A total of 75 participants (63.6%) reported that their programme explicitly assesses students' critical thinking. Most commonly, this occurred through the inclusion of critical thinking within assessment criteria, either embedded directly in assessment stimuli or articulated within marking rubrics (n = 62). Just under half also reported using student self‐ratings of their own critical thinking (n = 33). Only three participants indicated that their programme employed a formal, established assessment tool: Two reported using the Health Sciences Reasoning Test, and one reported using the Watson–Glaser Critical Thinking Appraisal.

At the individual teaching level, four participants reported not using any additional strategies to monitor students' critical thinking. The remainder described drawing on a range of informal and course‐embedded indicators, including students' performance in written assessments (n = 97) and verbal assessments (n = 82), their engagement during class activities (n = 90), their performance during practice education (n = 79), and/or feedback from practice educators (n = 65).

#### 3.2.4 Critical thinking at a programme and course level

All participants recognised critical thinking as essential for occupational therapy practice. However, there was variation in the extent to which it was valued and embedded within their programmes and courses. Although all participants reported that critical thinking was valued in their programmes to some degree, the perceived extent varied: 9.3% indicated it was valued to a small extent, 22.9% to a somewhat extent, 34.7% to a moderate extent, and 33.1% to a large extent. A consistent pattern emerged when participants were asked about the presence of the provided definition of critical thinking and its components of critical reflection, critical reason, and critical action within their programmes and courses. Across all components, a greater proportion of participants reported that their courses, compared with their programmes, featured these elements to a moderate or large extent.

Some significant differences were identified based on the survey language. Participants who completed the survey in Portuguese reported that the courses they taught incorporated the proposed definitions of critical thinking, critical reflection, critical reason, and critical action to a statistically higher degree than participants who responded in English (see Table 3). Similarly, Spanish responses indicated that the courses they taught featured critical thinking and action to a statistically higher degree than English responses. A statistically significant difference was also observed in relation to the extent to which critical action was represented within programmes, with Portuguese‐ and Spanish‐speaking participants again reporting higher levels than their English‐speaking counterparts (see Table 3).

### 3.3 Qualitative results

The following results are derived from the qualitative responses provided by participants. Table 4 presents the number of responses submitted for each qualitative question, categorised by language group. Notably, five of the six qualitative questions were mandatory.

**TABLE 4:** Number of qualitative responses by language group.

Questions	English	Portuguese	Spanish	Total	Percentage
Which subjects feature CT? They only had to provide list of subjects if they provide ‘YES’ that their subjects featured CT	46	20	14	80	67
CT is essential for OT	69	30	19	118	100
CT is useful for OT ed	69	30	19	118	100
How do you conceptualise CT?	68	30	19	117	99
Comments elaborating the ‘extent chosen’ regarding how much you agree with the definition of CT that was provided	36	30	15	81	68
Teaching methods used for CT	67	19	19	105	89

Abbreviations: CT, critical thinking; OT, occupational therapy.

#### 3.3.1 A spectrum of critical thinking

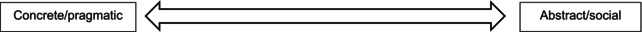

Participants discussed critical thinking in varied ways, reflecting a spectrum ranging from concrete, pragmatic, and individualistic aspects of occupational therapy to more abstract definitions linked to socially oriented dimensions. This spectrum was evident both in how participants described what critical thinking involves and in their articulation of its purpose. Some participants acknowledged both ends of this spectrum, whereas others framed critical thinking as situated primarily at one end. These differences were also reflected in responses to the proposed definition of critical thinking. For example, one participant noted that it was ‘a well elaborated definition’ (English‐language questionnaire group), whereas another stated, ‘I think it is a very complete definition that considers, to a large extent, many aspects that make critical thinking’ (Spanish‐language questionnaire group). However, three participants responding in English, despite agreeing to a moderate extent with the definition, expressed concerns that it was ‘too broad’, ‘confusing’, or would ‘need to be pared down to allow it to be more generalised’.

Across the dataset, references to concrete, pragmatic, practical, or individualistic aspects of critical thinking were most prominent. This perspective was most evident in the English‐language responses, which represented 59.3% of all survey submissions. In contrast, discussions of critical thinking that emphasised social dimensions of occupational therapy appeared more consistently in Portuguese‐language responses (22% of all responses). Spanish‐language responses (18.6% of all responses) also tended to emphasise this dimension of critical thinking, although many responses similarly incorporated concrete, pragmatic aspects of practice.

##### Concrete/pragmatic

At this end of the spectrum, critical thinking was described as ‘thoughtful and reasoned’ (English) and ‘systematic’ (Spanish) and as ‘the ability to carefully and respectfully analyse information that has been provided to make an informed decision’ (English). The types of information considered central to this process included ‘methods/interventions’ (English) and, more broadly, ‘factors in relation to person, environment and occupation’ (English). Many English‐language responses emphasised the role of evidence, identifying it as a key source of information to be critically evaluated. Others spoke generally about evaluating and synthesising ‘multiple perspectives’ (English).

Participants across all language groups discussed the purpose of critical thinking in similarly concrete and practice‐focused terms. These included making ‘informed decisions’ (Spanish), solving problems, supporting sound reasoning, and navigating research. Critical thinking was described as integral to selecting ‘which theoretical frameworks and models’ (Spanish) or types of intervention to use and to justifying the ‘why’ underpinning decisions and actions throughout the occupational therapy process. In doing so, it was viewed as essential for developing efficient, effective, and client‐centred practice: ‘to develop an efficient practice’ (Portuguese). Many participants, particularly those responding in English, explicitly framed these aspects of critical thinking within the language of ‘clinical reasoning’. However, references to clinical reasoning were also present in Spanish and Portuguese data. Evidence‐based practice (EBP) was similarly highlighted, primarily in English responses, as a ‘concept or maybe outcome of critical thinking’ (English). Some participants noted the absence of an explicit reference to EBP in the proposed definition of critical thinking and suggested that this omission reduced their agreement with the definition. Others perceived the definition to be overly theoretical, lacking an emphasis on application: ‘This definition is quite theoretical, does not seem to consider the skill in “applying” the critical thinking ability. This is a critical element to consider if we want occupational therapists to do this in clinical practice’ (English).

Participants across languages also described critical thinking as preventing unreflective or routine practice, helping avoid ‘technical knowledge without reflection, repetition of procedures, reductionist procedures, [and] recipes’ (Spanish). In this way, it was seen as instrumental to ensuring ‘individualised, client‐centred practice’ (English). The value of critical thinking for occupational therapy education mirrored many of these points. Participants emphasised its ‘importance for building clinical reasoning’ skills and for supporting students to ‘consider why we are approaching an aspect of the occupational therapy process in a certain way’ (English). The critical appraisal of research evidence, particularly in relation to client needs, was highlighted within English‐language responses as a key educational application of critical thinking.

##### Abstract/social

Participants also described critical thinking in ways that aligned with a more abstract definition, linked to socially oriented dimensions of occupational therapy practice. In these accounts, critical thinking involved ‘reflection’ and ‘questioning’, often situated within broader contextual, structural, or societal considerations. These perspectives were more frequently represented in Portuguese and Spanish responses. For example, participants highlighted the importance of ‘reflection on own doings in the context of the political, cultural, legal and economic institutions which frame our occupational therapy practice’ (English), ‘a deep reading about social reality and how it interferes in their [people's] life to act’ (Portuguese), and ‘the permanent reflection, questioning, contextualisation of theory and occupational praxis, linked to focusing on the subject to be treated within a social context’ (Spanish).

Within this theme, participants also identified knowledge, theory, and the profession itself as objects of interrogation within the critical thinking process. This notion appeared across all language groups but more prominently in Portuguese and Spanish responses. Participants described critical thinking as involving the questioning of ‘knowledge and professional roots’ (Spanish), the appraisal of ‘literature on occupational therapy and occupational science e.g. theories as well as values behind, and the practice of the profession’ (English), and ‘thinking (analysing) about thinking and considering biases, beliefs, knowledge … and the appraisal of ideologies and (various) contexts from where knowledge has been produced’ (English). These reflections were echoed in responses regarding the proposed definition of critical thinking, particularly among Portuguese and Spanish participants, who emphasised the importance of ‘problematising theory and practice’ (Spanish) and the connection between reflection and action. As one participant noted, ‘critical thinking … mobilises the subject to review his positions and his way of acting in the world, thus connecting reflection and action. That is why I agree with the definition provided’ (Portuguese).

A substantial group of participants described the purpose of critical thinking in terms of social transformation. Critical thinking was framed as essential ‘for emancipatory and situated praxis’ (Portuguese), to ‘transform reality and construct change’ (Spanish), and for ‘practice aimed at the liberation of the self and others from oppressive forces’ (English). This transformative orientation was related to occupational therapy education in terms of enabling ‘students to think more critically about their situatedness within social systems and structures and imagine possibilities to transform social practices’ (English), to ‘understand the everyday life, social problems, population level, situatedness of global issues’ (Spanish), and to ‘understand the indissociability between micro and macrosocial spheres’ (Portuguese). Although these perspectives were present across all language groups, they appeared more prominently in Portuguese and Spanish responses than in English ones. This orientation towards justice‐related practice was similarly articulated, particularly by Portuguese and Spanish speakers, who described critical thinking as vital for working ‘with the goal of developing autonomy, empowerment, emancipation with people in vulnerability, living unequal power relationships’ (Portuguese) and for ‘highlight[ing] injustices, inequalities, vulnerabilities, human rights, [and] visualiz[ing] political aspects of our practice, against the standardization of the everyday/occupations, neoliberalism’ (Spanish).

Variation across language groups was also reflected in reactions to the proposed definition of critical thinking. Spanish responses, and to a lesser extent English ones, expressed strong support for including social justice within the definition: ‘I argue that critical resides essentially not only in the desire for a less unequal world but also seeks practices aimed at achieving concrete transformations’ (Spanish). English‐language responses were the only ones to express uncertainty about this inclusion: ‘I am not sure within the occupational therapy community the ultimate goal is “social justice”’ (English).

At a more abstract level, participants also described the purpose of critical thinking as the interrogation of knowledge itself, aimed at pushing the boundaries of practice. Critical thinking was positioned as necessary ‘to avoid hegemonic models’ (Portuguese/Spanish), for ‘the deconstruction of colonial knowledge’ (Portuguese), for fostering ‘epistemological vigilance’ (Spanish), and to ‘question knowledge and professional roots’ (Spanish). English‐speaking participants also identified critical thinking as ‘the appraisal of ideologies and (various) contexts from where knowledge has been produced, so as to address the power and systemic injustices that have and do exist’ (English). This interrogation served to ‘not continue to do things the way they have always been done’ (English) and instead to ‘question current delivery of services and think critically to influence policy change’ (English). Such purposes were strongly reflected in participants' views on the role of critical thinking in occupational therapy education, where it was seen as crucial for ‘questioning our knowledge versus naturalization of practices’ (Spanish) and for fostering recognition of the limitations of knowledge, including developing skills ‘in assessing the nature and value of knowledge presented, and assist[ing] to challenge universal truths’ (English).

Within the proposed definition of critical thinking, this orientation towards transformation was captured within the component of critical action. Many participants across all language groups expressed strong support for including action as an integral part of critical thinking, though this view was especially pronounced in the Portuguese responses: ‘I agree that thought does not stop at thought but manifests itself in action’ (Portuguese). English‐language responses also reflected support for this inclusion, noting that the definition ‘moves beyond superficial critical thinking and encourages people to change practice and the processes around them’ (English). Another participant explicitly connected this reflection–action relationship to Freirean praxis, stating the following: ‘I believe the relationship between reflection‐action (Freirean Praxis) is necessary both for continuous training of our discipline and in the impact and engagement with the people, groups and communities with whom we work’ (Portuguese). English responses were the only ones to voice apprehension about the appropriateness of including action within the definition of critical thinking: ‘I would see critical action as ideal, but I see it outside the definition of “critical thinking”’ (English).

##### Teaching the spectrum of critical thinking

Consistent with the broader spectrum of critical thinking described by participants, most respondents reported that critical thinking was explicitly taught across a wide range of courses within their occupational therapy programmes. These included subjects such as ‘epistemology, methodology, evidence‐based practice, professional reasoning, and critical thinking based on social sciences readings’ (English). Across the dataset, the most prominent group of subjects in which participants reported explicitly teaching critical thinking was those related to clinical or professional reasoning and foundational occupational therapy courses. These included ‘occupational therapy practice topics where critical thinking is taught in relation to practice and applied clinical decision making’ (English) and ‘occupation and professional reasoning’ (Spanish). For some participants, these were the *only* subjects in which they identified explicit teaching of critical thinking.

Another group of participants, primarily in the English‐language responses, listed research‐related courses such as ‘biostatistics and research methodology’ (English) as the central spaces where critical thinking was taught. EBP subjects were also mentioned exclusively within the English data. For some English‐speaking participants, research and EBP courses were the only courses identified as explicitly teaching critical thinking. By contrast, research‐related subjects had far less prominence in the Spanish and Portuguese responses.

A further cluster of responses highlighted subjects that explore broader social, cultural, and structural dimensions of practice, such as anthropology, sociology, and public health, described in one instance as courses that examine ‘the societal context of occupation’ (English). These courses were most strongly represented within the Portuguese responses. Participants elaborated on specific topics within these subjects, including ‘culture and occupational therapy’ (Portuguese), ‘human rights, socio‐cultural diversity and minorities, vulnerable populations, poverty, unemployment, solidarity …’ (Portuguese), and ‘occupation and socio‐political context’ (Spanish). These subjects reflect the more abstract definition, linked to the social end of the critical thinking spectrum.

## 4 DISCUSSION

This study explored how occupational therapy educators across linguistic and geographical contexts conceptualise and teach critical thinking and how it is featured in the programmes they teach. The findings demonstrate a spectrum of critical thinking, ranging from concrete, pragmatic, and practice‐oriented understandings to abstract, socially oriented, and transformative conceptualisations. A key contribution of this study is the identification of epistemological variation in how critical thinking is understood. English‐language responses tended to emphasise concrete and pragmatic aspects of critical thinking, aligning with clinical reasoning, evidence appraisal, and decision‐making. These patterns reflect broader debates in health‐care education, where definitions and teaching methods for critical thinking are often linked to technical rationality and EBP frameworks (Berg et al., 2023; Pitonyak et al., 2020). They also embody the prevailing epistemologies found in many Western occupational therapy curricula, where positivist and individual‐focused reasoning traditions continue to shape pedagogical approaches. Literature reviews similarly highlight variability in methodologies and inconsistent outcomes in critical thinking instruction, underscoring the lack of shared understandings (Behar‐Horenstein & Niu, 2011; Berg et al., 2023).

In contrast, Portuguese and Spanish responses more frequently articulated abstract definitions, linked to socially situated perspectives. Respondents emphasised critical reflection on knowledge production, ideological assumptions, social structures, and the political dimensions of occupation, as well as the more prominent featuring of critical action in their curricula. These perspectives resonate with longstanding calls in occupational therapy and occupational science to engage with structural conditions, rights, and critical praxis, including reflection–action linkages central to transformative practice (Farias & Lopes, 2022; Farias & Rudman, 2019a; Restall, 2024; Schiller et al., 2023). Latin American occupational therapy, particularly in Brazil, has a robust history of community‐based, critical, and socially oriented practice (Lopes & Malfitano, 2021). Consequently, it is unsurprising that educators in these contexts emphasised critical action, social transformation, and questioning of hegemonic knowledge. This emphasis aligns with international arguments for building contextually grounded occupational therapy that moves beyond the dominance of Eurocentric theoretical frameworks (Hammell, 2019; Mahoney & Kiraly‐Alvarez, 2019).

Although language differences contribute to variation in how concepts are expressed, the findings suggest that language alone does not account for the observed epistemological variation. Rather, the language groups represent broader historical, political, and professional contexts that shape how critical thinking is understood and taught. In Anglophone contexts, occupational therapy scholarship has often been shaped by Euro‐Western traditions that prioritise technical rationality and efficiency. Critics have urged the profession to interrogate the universality of these assumptions and to diversify its theoretical base (Farias & Rudman, 2019b; Hammell, 2019). Conversely, Portuguese‐ and Spanish‐speaking contexts often foreground collective (Firsenko & Malfitano, 2025), community‐based, and emancipatory approaches, resonating with scholarship emphasising social participation, structural inequalities, and the political nature of everyday life (Godoy‐Vieira et al., 2024). These interpretations also align with wider debates in internationalised higher education that highlight sociopolitical engagement and global citizenship as essential graduate capabilities (Kraska et al., 2018). Collectively, these results reflect epistemological diversity within the profession, rather than mere linguistic variation, highlighting divergent assumptions about what counts as knowledge, what forms of reasoning are valued, and the purposes critical thinking serves in occupational therapy practice. They also mirror uncertainties highlighted in the higher education literature regarding cultural assumptions embedded in critical thinking expectations (Liyanage et al., 2021).

A central question arising from the findings is whether the absence or underrepresentation of abstract and critical epistemologies in many English‐language responses could pose a risk for the conceptualisation and teaching of critical thinking. We argue that the challenge is not the presence of diverse epistemologies per se; rather, overreliance on any single epistemological stance restricts the profession's capacity to address the complex cultural, social, and political challenges shaping contemporary practice. Calls within occupational therapy education to clarify critical thinking and distinguish it from clinical reasoning underscore this risk and the need for a broader, more explicit pedagogical orientation (Gilfillan et al., 2024). Rather than prescribing a universal definition, our findings point to the benefits of broadening the profession's conceptualisation to encompass critical reflection, critical reason, and critical action.

Occupational therapy has long been critiqued for its Eurocentrism and Global North centrism (Taff & Putnam, 2022), its alignment with Western biomedical frameworks, and its limited engagement with structural inequities. Expanding conceptualisations of critical thinking to include critical epistemologies supports a more inclusive, reflexive, and socially responsive profession. It also aligns with emerging calls to recognise that diverse ways of knowing, being, and doing are attuned to the sociopolitical realities shaping practice (Restall, 2024).

### 4.1 Strengths and limitations

The use of a mixed‐methods design allowed a thorough exploration of quantitative patterns alongside detailed qualitative insights. Collecting data in three languages (i.e., English, Spanish, and Portuguese) expanded the study's international scope and facilitated examination of epistemological diversity across various linguistic and cultural contexts, making a significant contribution to occupational therapy education research. Rigour was enhanced through first‐language analysis, collaborative cross‐language comparison, and ongoing reflexive engagement within an international research team.

Regarding limitations, some participants might have completed the survey in a second or third language, which could have restricted the depth or accuracy of their responses. Limiting the survey to three languages may also have excluded potential participants. Additionally, participants were asked to comment on their programmes, but not all may have had comprehensive knowledge of every curriculum component, which could have contributed to differences observed between programme‐ and course‐level responses. Lastly, voluntary participation might have attracted educators already interested in critical thinking, possibly influencing the prominence of certain conceptualisations.

## 5 CONCLUSIONS

The epistemological diversity identified in this study offers valuable insights into the multiplicity of ways in which occupational therapy educators conceptualise critical thinking. Rather than striving for epistemological uniformity, the profession stands to benefit from embracing this diversity. Recognising and critically examining the epistemological underpinnings that shape our conceptualisations of critical thinking, and the curricular purposes they serve, can support a more inclusive and socially responsive profession attuned to contemporary complexities.

## AUTHOR CONTRIBUTIONS

**Laura Irvine‐Brown**: Conceptualization; formal analysis; methodology; investigation; writing—original draft; writing—review and editing. **Ana Malfitano**: Formal analysis; investigation; writing—review and editing. **Shawna Mastro Campbell**: Formal analysis; methodology; writing—review and editing. **Amelia Di Tommaso**: Conceptualization; methodology. **Lisette Farias**: Formal analysis; investigation; writing—original draft; writing—review and editing.

## CONFLICT OF INTEREST STATEMENT

The authors have no conflicts of interest to declare.

## DECLARATION OF USE OF ARTIFICIAL INTELLIGENCE

The authors have not used artificial intelligence in the writing of this manuscript.

## ETHICS APPROVAL

The study had ethical approval from the Griffith University Ethics Committee (GU Ref No. 2021/915).

## Data Availability

The data that support the findings of this study are available on request from the corresponding author. The data are not publicly available due to privacy or ethical restrictions.
